# Seasonal Variation of Carbon Metabolism in the Cambial Zone of *Eucalyptus grandis*

**DOI:** 10.3389/fpls.2016.00932

**Published:** 2016-06-28

**Authors:** Ilara G. F. Budzinski, David H. Moon, Pernilla Lindén, Thomas Moritz, Carlos A. Labate

**Affiliations:** ^1^Laboratório Max Feffer de Genética de Plantas, Departamento de Genética, Escola Superior de Agricultura Luiz de Queiroz, Universidade de São PauloPiracicaba, Brazil; ^2^Umeå Plant Science Centre, Department of Forest Genetics and Plant Physiology, Swedish University of Agricultural SciencesUmeå, Sweden

**Keywords:** *Eucalyptus grandis*, primary metabolism, RT-qPCR, 2-DE Proteome, metabolome, ethanolic fermentation

## Abstract

Eucalyptus species are the most widely hardwood planted in the world. It is one of the successful examples of commercial forestry plantation in Brazil and other tropical and subtropical countries. The tree is valued for its rapid growth, adaptability and wood quality. Wood formation is the result of cumulative annual activity of the vascular cambium. This cambial activity is generally related to the alternation of cold and warm, and/or dry and rainy seasons. Efforts have focused on analysis of cambial zone in response to seasonal variations in trees from temperate zones. However, little is known about the molecular changes triggered by seasonal variations in trees from tropical countries. In this work we attempted to establish a global view of seasonal alterations in the cambial zone of *Eucalyptus grandis* Hill ex Maiden, emphasizing changes occurring in the carbon metabolism. Using transcripts, proteomics and metabolomics we analyzed the tissues harvested in summer-wet and winter-dry seasons. Based on proteomics analysis, 70 proteins that changed in abundance were successfully identified. Transcripts for some of these proteins were analyzed and similar expression patterns were observed. We identified 19 metabolites differentially abundant. Our results suggest a differential reconfiguration of carbon partioning in *E. grandis* cambial zone. During summer, pyruvate is primarily metabolized via ethanolic fermentation, possibly to regenerate NAD^+^ for glycolytic ATP production and cellular maintenance. However, in winter there seems to be a metabolic change and we found that some sugars were highly abundant. Our results revealed a dynamic change in *E. grandis* cambial zone due to seasonality and highlight the importance of glycolysis and ethanolic fermentation for energy generation and maintenance in *Eucalyptus*, a fast growing tree.

## Introduction

*Eucalyptus* is the most widely planted hardwood genus in the world. The trees are valued for their fast growth, high adaptability to different climatic conditions and multiple uses of their wood (e.g., pulp and paper industries, charcoal-based steel, wood panels and potential feedstock for lignocellulosic biofuels; Albaugh et al., 2013; Nogueira et al., 2015). Wood formation (xylogenesis) is a complex and highly dynamic process. It is the result of cumulative annual activity of the vascular cambium (Li et al., 2010), a secondary meristem which repeatedly induces its cell division, originating xylem and phloem and is responsible for self-maintenance and signal transfer via translocation of growth regulators (Larson, 1994). The annual course of cambial activity is generally related to the alternation of cold and warm, and/or dry and rainy seasons (Lachaud et al., 1999). The knowledge about cambial activity is fundamental since it is the time in which trees receive environmental signals directly responsible for their growth (Callado et al., 2014). Perennial woody plants from temperate zones have developed mechanisms, that undergo seasonal cycles of activity and dormancy, which are collectively known as annual periodicity (Ko et al., 2011; Begum et al., 2013). This periodicity plays an important role in the formation of wood and reflects the environmental adaptation of woody species. Therefore, the quantity and quality of wood depend on the division of cambial cells and the differentiation of cambial derivatives (Begum et al., 2013). In tropical regions (e.g., Brazil) seasonal changes are less pronounced than in temperate regions, cambial activity is relatively longer and may continue throughout the year (Prislan et al., 2013). It is suggested that in tropical regions water availability is the main factor that induces cambial seasonality. An annual dry season with a length of 2–3 months and less than 60 mm monthly precipitation induces the reduction in cambial activity, which is reestablished in the seasons where monthly precipitations are higher (Worbes, 1995). Oliveira et al. (2012) investigated the relationship between precipitation and wood production in a 23-years old *Eucalyptus grandis* in Brazil, using x-ray densitometry. The results showed a positive correlation between precipitation data and annual increment of wood. The molecular and physiological mechanisms that enable trees to survive and maintain themselves under limiting conditions, such as winter and limited water availability, are crucial to woody plants (Ko et al., 2011). However, the molecular mechanisms that occur in the cambial zone during seasonal changes are largely unknown. Furthermore, despite the influence that seasonality has on cambial activity, molecular studies regarding the changes that occur in these tissues have only been carried out in species from temperate zones, emphasizing cold acclimation (Schrader et al., 2004; Yang et al., 2004; Gricar and Cufar, 2008; Li et al., 2010; Ko et al., 2011; Galindo-González et al., 2012).

Glycolysis and Tricarboxylic Acid (TCA) cycle are known as central backbones of plant primary metabolism. Under aerobic conditions, pyruvate is transported into mitochondria and oxidized through TCA cycle into organic acids, CO_2_ and water, via aerobic respiration. During these steps the reducing equivalent, NADH is formed and used by the mitochondrial electron transport chain to power the synthesis of ATP (Fernie et al., 2004). However, under oxygen-limiting conditions (hypoxia), fermentative metabolism is activated to recycle NAD^+^ from NADH in order to avoid depletion of the cytosolic NAD pool (Zabalza et al., 2009) and to keep glycolysis running in the absence of oxidative phosphorylation by the mitochondrial electron transport chain (van Dongen et al., 2011). Oxygen availability is a key factor in respiration metabolism because it is the final electron acceptor in the oxidative phosphorylation (Moller, 2001). However, under oxygen-limiting conditions (hypoxia), plants have developed alternative pathways to aerobic respiration (e.g., fermentative reactions) in order to maintain glycolysis through the regeneration of NAD^+^ and thus obtaining the energy necessary to maintain key process of metabolism (membrane integrity, protein synthesis and turnover, regulation on cytosolic pH, among others (Tadege et al., 1999; Rocha et al., 2010). Thus, pyruvate might be converted to ethanol by pyruvate decarboxylase (PDC) and alcohol dehydrogenase (ADH; Zabalza et al., 2009). In trees from tropical areas this hypoxic condition is probably more intense during summer, when trees (e.g., *Eucalyptus*) are growing fast. Moreover, it is known that the bark has a low permeability to gaseous diffusion (Waisel, 1995; Sorz and Hietz, 2006). The reduced bark permeability may limit the atmospheric oxygen diffusion to the cambium zone, causing a reduction in respiration, by both, limiting the regenerating capacity of NAD^+^ and increasing the accumulation of CO_2_ (Pfanz et al., 2002; Aschan and Pfanz, 2003). Such changes in primary metabolism could lead to a reconfiguration of carbon allocation during summer and winter. Celedon et al. (2007) and Carvalho et al. (2008) reported the identification of proteins and transcripts related to carbon metabolism in the cambial zone of *E. grandis* trees at different ages, including alcohol dehydrogenase and pyruvate decarboxylase. Leonardi et al. (2015) identified stem proteins (e.g., energy metabolism) potentially related to cold stress responses in *Eucalyptus urograndis*. On the other hand, Durand et al. (2011) found two sucrose synthase more abundant in the cambial zone of *Populus tremula* L. × *P. alba* L. submitted to drought stress.

Given the importance of *Eucalyptus* wood to tropical and subtropical regions, the objective of the present study was to identify changes in transcripts, proteome and metabolome that occur in *E. grandis* cambial zone during summer (wet season and cambial active growth) and winter (dry season). We hypothesize that during the summer season, the rapid growth and intense metabolic activity in *E. grandis* trees is compensated by an increase in glycolysis and ethanolic fermentation, in order to maintain the carbon metabolism in the cambial zone. To our knowledge this is the first study of cambial zone metabolism analyzed at three different molecular levels for a tropical and subtropical cultivated tree. This approach allows for a more comprehensive characterization of the metabolic processes that are occurring during summer and winter. We found significant changes in the metabolism of the cambial zone, suggesting that during summer, pyruvate produced by glycolysis is primarily diverted to ethanolic fermentation, to regenerate NAD^+^ and maintain glycolysis producing ATP. However, during winter we found some sugars and transcripts related to sugar biosynthesis highly abundant. We also hypothesized that these metabolic changes between summer and winter are mainly triggered by higher water availability during the fast growing period contrasting with diminished water in the dry season (winter). Taken together, these results show that different pathways are actively expressed in summer/winter tree and provide new insights into molecular basis of cambial zone differential responses to seasonality in tropical trees.

## Materials and methods

### Plant material and experimental conditions

Tissue samples were harvested from clonal trees of 6 years-old *E. grandis* W. Hill ex Maiden, kindly provided by Suzano Papel e Celulose. The field-trial was situated in Itapetininga, State of São Paulo, Brazil (23°35′20″ S, 48°03′11″ W) at an altitude of 656 m. The cambial zone of the trees was harvested during the summer (26/January/2009), the actively growing season and during the winter (19/August/2009), less-actively growing season. The term cambial zone adopted here describes the entire group of cells which includes cambial initials, xylem and phloem mother cells and developing tissues (differentiating xylem and phloem). To collect the cambial zone, bark of each tree was removed at chest height (1.30 m, exposing an area of ~20 × 15 cm) and the differentiating phloem and xylem were scraped with a razor blade from the inner side of the bark and the outermost side of the stem, respectively, as shown in Supplementary Figure 1. Tissues harvested were immediately frozen in liquid nitrogen. The stem tissue was scraped until the fibrous material below the differentiating cells was reached as described by Celedon et al. (2007) and Carvalho et al. (2008). Samples were harvested in the morning, between 9 a.m. and 10 a.m. The average temperature and precipitation data based on the previous 15 days before sampling were 22.5°C and 99.0 mm, respectively, in summer/January 2009. During the month of winter/August 2009 these parameters were 16.8°C and 15.3 mm, respectively. The field-trial was a completely randomized design. Six bulked samples (10 trees each) were made by grinding and mixing the sampled material. Three bulks represented summer and three represented winter trees.

### RNA extraction and mRNA isolation

Total RNA was extracted from the cambial zone tissue using the protocol described by Zeng and Yang (2002). Total RNA concentration was measured spectrophotometrically at 260/280 nm, using a U-3300 spectrophotometer (Hitachi, Tokyo, Japan). The absence of RNA degradation was verified by electrophoresis on a formamide-formaldehyde denaturing agarose gel (1%). mRNA was isolated from total RNA (50 μg) using Dynabeads® mRNA purification kit (Invitrogen Dynal, Oslo, Norway), according to the manufacturer's instructions.

### RT-qPCR

Gene-specific primers pairs were designed with the Primer 3 software (Supplementary Table 1). Primer pairs were designed as follows: primer length between 18 and 25 bp, product length of 100–250 bp, melting temperatures 55–60°C, GC% between 40 and 60%. First and second strand cDNA synthesis were performed using the *SuperScript*™*One-Step RT-PCR Platinum® Taq* kit (Invitrogen, Carlsbad, CA, USA) with RT/Platinum® *Taq* (Invitrogen, Carlsbad, CA, USA) and using primers specific for the genes of interest (Supplementary Table 1). The cDNAs were produced in a Gene Amp® PCR System 9700 thermocycler (Applied Biosystems, Foster City, CA, USA) using as annealing temperatures 57°C. The cDNAs were used as a template for RT-qPCR assays, carried out in an iQ5 instrument (BioRad) to obtain de threshold quantification cycle (*Cq*) and the amplification efficiencies (*E*). At the end of the PCR cycles, the thermocycler was programmed to perform a denaturation curve. The final volume of each reaction was 20 μL, including cDNA, 10 mM of each primer and 1x Supermix SYBR Green real-time RT-PCR (Invitrogen). The reaction condition was as follows: 95°C for 30 s, 35 cycles of 95°C for 10 s, 57°C for 10 s, and 72°C for 10 s. A negative control (no cDNA template) was included for every gene. All RT-qPCR reactions were carried out in triplicate. The software *LinReg* (Ramakers et al., 2003) was used to calculate the PCR efficiencies and the C_*q*_ values of each gene analyzed. Reference genes were identified using *NormFinder* (Andersen et al., 2004) which gave the best stability value (0.4) for the combination of alpha-tubulin and malate dehydrogenase (*MDH*) genes. The calculation of relative expression ratios was carried out with the Relative Expression Software Tool (REST) using the pairwise fixed reallocation randomization test for the statistical significance (*P* ≤ 0.05; Pfaffl et al., 2002).

### Protein extraction and IEF-SDS PAGE

Total protein of the cambial zone was extracted from 6 grams of frozen ground tissue using the phenolic method according to Hurkman and Tanaka (1986), with minor modifications described in Celedon et al. (2007). The protein pellet was dried under vacuum at 4°C and suspended in 1 mL of solubilization buffer [7 M urea, 2 M thiourea, 0.4% v/v Triton X-100, 50 mM dithiothreitol (DTT)]. Proteins were quantified using the Bradford method (Bradford, 1976). Protein samples (500 μg) were mixed with buffer (340 μL) containing 10 mM DTT, 4% (w/v) CHAPS, 1% IPG buffer (GE Healthcare, Chalfont St. Giles, UK) and 1% (w/v) bromophenol blue and used to rehydrate Immobiline IPG strips (18 cm, 4–7 linear immobilized pH, GE Healthcare, Chalfont St. Giles, UK) for 12 h at 20°C using 50 V. Rehydrated strips were isoelectrofocused (IEF) in an Ettan™IPGphor II™(GE Healthcare) at 100 V for 1 h and then 500 V for 1 h, 1000 V for 1 h, 5000 V for 1 h, and 8000 until reaching a total of 80000 V-h. After IEF, the strips were kept at −80°C until needed. Before the second dimension, strips were kept at room temperature for 15 min in equilibration buffer (6 M urea, 2% w/v SDS, 50 mM Tris-HCl, pH 6.8, 30% v/v glycerol) firstly, with 1% w/v DTT and then with 2.5% w/v iodoacetamide (IAA) and 0.001% bromophenol blue. The second dimension was performed in 12% (w/v) polyacrylamide gels, using Protean II XI 2-D cell electrophoresis system (GE Healthcare), at 30 mA per gel. Three biological replicates were performed for each treatment. Proteins were detected using Coomassie Brilliant Blue G-250 (Candiano et al., 2004), with modifications. Gels were incubated for 1 h in a solution containing 40% (v/v) ethanol and 10% (v/v) acetic acid, in water. For protein detection the gels were left overnight in staining solution (20% (v/v) methanol, 10% (w/v) ammonium sulfate, 10% v/v phosphoric acid, and 0.1% (w/v) Coomassie G-250). After three washes in water (2 h each), the gels were stored in 5% (w/v) ammonium sulfate until image analysis, spot selection and picking.

### Gel image analysis

Gels were imaged using Image scanner III and Labscan v 7.0 software (GE Healthcare). Image analysis was performed automatically using the Image Master 2D Platinum software v 7.0 (GE Healthcare). Gels from three independent biological replicates were used. Spots were detected using a smoothness of 8, minimum area of 15 and a saliency of 40, and spots across gels were matched using 5 landmarks per gel. Matching was performed automatically and systematically confirmed after one-by-one visual checking: artifacts, or spots that could not be confidently validated as true matches were disregarded and misalignments were corrected manually when appropriate. Spots were considered reproducible when well-resolved in at least two of the three biological replicates. The abundance of each protein spot was estimated by the percentage of volume (% vol). For each treatment analyzed the average spot volume of the three replicate gels was determined, followed by normalization (individual spot volume/total spot volume × 100). The normalized volumes (% vol.) of the corresponding spots from summer and winter samples were compared to estimate differential expression of proteins during different seasons. To ensure the reproducibility between biological replicates, spots with coefficient of variation higher than 30% were excluded. To identify spots that changed in abundance between the two seasons, the data collected from protein spots volumes were subjected to Students *t-test* (*P* ≤ 0.05) in Image Master v 7.0 software. The fold-change (summer/winter) value of each identified spot were also given (Table 1). However, no fold-change cut-off was established and their values were not taken into account in data discussion.

**Table 1:** **Identification of proteins spots that changed in abundance from 2-DE gels (***P*** ≤ 0.05)**.

**Spot n°**	**Protein name**	**Protein Score**	**N° of Peptides**	**Coverage %**	**Sequence**	**Fold Change (summer/winter)**	***P*-value**
**1.1.2 ENERGY METABOLISM (CARBON)**							
3	Transketolase	1451	6	32	Eucgr.D02466.1	2.48	1.2E-04
4	Fructose-bisphosphate aldolase	676	2	47	Eucgr.K02073.1	5.94	1.6E-04
5	Alcohol dehydrogenase 1	378	3	19	Eucgr.F02744.1	4.45	1.8E-04
6	Fructose-bisphosphate aldolase 2	256	2	23	Eucgr.K02073.1	4.46	2.6E-04
17	Alcohol dehydrogenase 2	149	3	21	Eucgr.I00224.2	2.44	2.2E-03
22	Glyceraldehyde-3-phosphate dehydrogenase C	1459	3	62	Eucgr.B00144.1	0.71	3.4E-03
27	Phosphoglycerate kinase	3180	7	61	Eucgr.F04463.1	2.16	4.7E-03
29	alpha/beta-Hydrolases superfamily protein	167	6	8	Eucgr.D02325.2	1.57	5.2E-03
48	Alcohol dehydrogenase 3	123	3	40	Eucgr.I00224.2	0.27	1.1E-02
55	Pyruvate decarboxylase	1043	3	28	Eucgr.A00549.1	1.66	1.6E-02
88	Phosphoglucose isomerase 1	313	4	19	Eucgr.F02133.1	0.34	3.1E-02
**1.1.3 ENERGY TRANSFER/ATP-PROTON MOTIVE FORCE**							
18	Cytosolic NADP+-dependent isocitrate dehydrogenase	651	8	22	Eucgr.F02901.1	4.62	2.3E-03
19	Citrate synthase	70	4	11	Eucgr.G03412.1	2.98	2.1E-03
30	Adenylyl Cyclase associated protein 1	146	4	16	Eucgr.I01265.1	1.98	5.3E-03
33	ATP synthase alpha/beta family protein	228	7	18	Eucgr.G02224.1	2.07	6.2E-03
47	Quinone oxidoreductase	1700	13	58	Eucgr.I01801.3	1.7	1.0E-02
53	Pyruvate dehydrogenase complex E1 alpha subunit	346	5	24	Eucgr.B03379.1	2.47	1.4E-02
**1.2.1 AMINO ACID METABOLISM**							
15	Methyl-thioribose kinase	389	5	18	Eucgr.I01098.1	2.49	1.9E-03
26	5-methyltetrahydrofolate	1132	6	24	Eucgr.K01508.1	5.3	4.4E-03
67	Methylene tetrahydrofolate reductase2	367	10	26	Eucgr.A00394.1	3.56	2.0E-02
**1.2.3 NUCLEOTIDE/NUCLEOSIDE AND NUCLEOTIDE SUGAR METABOLISM**							
12	UDP-glucose pyrophosphorylase2	2173	3	43	Eucgr.F02905.1	2.73	1.2E-03
25	UDP-sugar pyrophosphorylase	187	8	25	Eucgr.F03856.1	2.32	3.9E-03
63	Adenosine kinase	1234	8	47	Eucgr.K01249.1	1.41	1.7E-02
**1.2.4 PHOSPHORUS METABOLISM**							
89	Pyrophosphatase	276	2	18	Eucgr.A01238.1	2.56	3.2E-02
**1.2.8 SECONDARY METABOLISM**							
11	Isoflavone reductase	698	12	69	Eucgr.E00336.1	2.79	1.2E-03
23	Chalcone isomerase like-protein	168	2	16	Eucgr.G03138.2	0.76	3.5E-03
34	Chalcone and stilbene synthase family protein	81	4	12	Eucgr.H02828.1	2.9	6.3E-03
51	Isoflavone reductase-like	1983	3	80	Eucgr.E00336.1	0.7	1.3E-02
72	Anthocyanidin synthase	184	4	22	Eucgr.D01945.1	0.77	2.3E-02
100	Chalcone-flavanone isomerase family protein	73	2	14	Eucgr.G03138.2	1.91	4.9E-02
**2.2.2 PROTECTION RESPONSES/DETOXIFICATION**							
8	Ascorbate peroxidase	1207	6	36	Eucgr.B02456.1	0.61	4.8E-04
**3.1.1 PROTEIN, PEPTIDE TRANSPORT**							
60	Importin alpha isoform	465	3	19	Eucgr.H03281.1	1.63	1.7E-02
**3.4 TRANSPORT FACTORS, KINESINS**							
106	Nuclear transport factor	70	1	10	Eucgr.H04629.1	0.33	2.0E-05
**4.1.2.4 LIGNIN METABOLISM**							
16	Caffeic-O-methyltransferase1	778	6	39	Eucgr.A01397.1	1.74	2.2E-03
37	Caffeic-O-methyltransferase1	1671	9	38	Eucgr.A01397.1	1.48	6.8E-03
92	Caffeoyl-CoA O-methyltransferase2	150	4	14	Eucgr.G01417.1	2.7	3.5E-02
**4.1.2.5 EXPANSINS, XET, AND EXTENSIN**							
102	Major allergen pru	348	3	26	Eucgr.A00159.1	0.46	5.8E-03
**4.3 CYTOSKELETON**							
9	Tubulin alpha-3	647	8	25	Eucgr.B03604.1	5.15	5.3E-04
103	Tubulin folding cofactor A	114	1	18	Eucgr.E01748.1	0.63	8.7E-02
74	Tubulin alpha-5	131	4	10	Eucgr.A02108.1	2.64	2.4E-02
**4.5 CHROMOSOME RELATED**							
52	14-3-3 protein	173	4	16	Eucgr.F02130.1	0.49	1.3E-02
**5.2 RNA RELATED**							
108	Serine threonine protein phosphatase	75	1	10	Eucgr.K02214.1	0.55	3.5E-02
**5.3.3 TRANSLATION RELATED**							
35	Elongation factor 1-gamma	385	4	13	Eucgr.I02782.1	2.86	6.4E-03
**5.3.4 PROTEIN MODIFICATION/PHOSPHORYLATION**							
45	Serine threonine-protein kinase	134	5	25	Eucgr.A01018.1	1.76	9.7E-03
73	Ribosomal protein S5/Elongation factor G/III/V protein	1398	9	35	Eucgr.F01462.2	2.19	2.4E-02
80	Protein phosphatase 2A subunit A2	664	3	31	Eucgr.B03031.1	2.14	2.7E-02
**5.3.5 PROTEINFOLDING/CHAPERONING**							
24	Heat shock protein 70	728	12	25	Eucgr.J00025.1	2.09	3.5E-03
31	ATP-dependent Clp protease	153	8	12	Eucgr.K02198.2	2.27	5.9E-03
39	Heat shock protein 70	376	8	14	Eucgr.J02987.2	2.37	6.9E-03
40	ATPase, AAA-type, CDC48 protein	1865	8	39	Eucgr.K01258.1	1.3	7.1E-03
41	ATPase, AAA-type, CDC48 protein	128	3	5	Eucgr.K01258.1	2.26	7.3E-03
43	TCP-1/cpn60 chaperonin family protein	321	3	18	Eucgr.B03239.1	7.05	8.1E-03
49	TCP-1/cpn60 chaperonin family protein	1522	8	46	Eucgr.J00618.1	3.32	1.2E-02
54	ATPase, AAA-type, CDC48 protein	993	10	26	Eucgr.K01256.1	2.05	1.5E-02
57	Regulatory particle triple-A ATPase 3	1038	6	34	Eucgr.B04032.1	2.26	1.7E-02
59	Heat shock protein 70B	87	9	14	Eucgr.F03980.1	0.77	1.7E-02
68	Heat shock protein	187	4	7	Eucgr.A01044.1	2.04	1.2E-02
79	Heat shock 70B	608	5	27	Eucgr.J03127.1	2.23	2.5E-02
83	TCP-1/cpn60 chaperonin family	151	7	16	Eucgr.B03239.1	0.31	2.8E-02
91	Luminal binding protein	1161	12	23	Eucgr.E01024.1	2.23	3.4E-02
94	Heat shock protein 70 B	828	5	17	Eucgr.E01024.1	0.45	3.8E-02
99	Heat shock protein 70 family protein	1092	3	30	Eucgr.J02987.2	1.38	4.9E-02
105	IV Heat shock protein	193	3	11	Eucgr.F04109.1|	0.5	3.2E-03
110	HSP20-like chaperones super family	838	3	48	Eucgr.J01957.1	0.46	1.7E-02
**5.3.6 PROTEIN CLEAVAGE AND TURNOVER**							
76	CLPC homolog 1	469	9	21	Eucgr.K02198.2	1.55	2.5E-02
87	Chalcone isomerase-like protein	374	3	22	Eucgr.G03138.1	1.44	3.0E-02
**6.2 PUTATIVE PROTEIN**							
28	Protein of unknown function (DUF674)	1016	3	19	Eucgr.J00059.2	1.53	4.8E-03
64	Probable nucleoredoxin 1-like	81	2	5	Eucgr.H02592.1	3.02	1.8E-02
70	Protein usf-like	217	6	28	Eucgr.K00785.1	1.61	2.2E-02
86	Proteasome subunit alpha type-2-a-like	93	5	22	Eucgr.F04120.1	0.37	3.0E-02

### Protein identification by mass spectrometry

In-gel digestion of proteins was done as described in Celedon et al. (2007). After, peptide mixtures were sequenced by online chromatography using a nano-Acquity UPLC (Waters®) system coupled to a Q-TOF Ultima API mass spectrometer (Waters, UK). Mass spectrometer parameters were set according to Celedon et al. (2007). Ten microliters of sample were loaded onto a pre-column Symmetry C18 5 μm, 5 × 30 mm (Waters) for sample preconcentration and desalination, followed by peptide separation on an LC column Symmetry C18, 5 μm, 32 × 150 mm (Waters). Peptides were eluted using a10–45% linear gradient of solvent B [95% (v/v) ACN, 0.1% (v/v) FA in water], starting at a flow rate of 5 μL/min for the first 15 min, then changing to 2 μL/min for the next 25 min, and back to 5 μL/min in the last 5 min. Solvent A consisted of 5% v/v ACN, and 0.1% v/v FA in water. All analyses were performed using a positive ion mode at 3 kV needle voltage. The mass range was set from *m/z* 300 to 2000, and the MS/MS spectra were acquired for the most intense peaks having at least 15 counts. The LC-MS/MS were processed using ProteinLynx v 2.0 (Waters) and Mascot Daemon software (Matrix Science, Boston, MA), and the sequences searched against an in-house *Eucalyptus* database from Phytozome v1.1 (www.phytozome.net/eucalyptus.php) and NCBI. Combined MS/MS search criteria used were as follows: trypsin digestion; fixed modification set as carbamidomethylation of cysteine; variable modification set as methionine oxidation; mass accuracy of 50 ppm for the parent ion and MS/MS mass tolerance of 0.1 Da. According to MASCOT probability analysis, only significant hits (*P* ≤ 0.05) were accepted. A match was considered significant if the peptide had a score higher than 70, based on Perkins et al. (1999). The mass spectrometry proteomics data have been deposited to the ProteomeXchangeConsortium (http://proteomecentral.proteomexchange.org) via the PRIDE partner repository (Vizcaino et al., 2013) with the dataset identifier (PXD003504).

### Metabolite extraction, data processing, and statistical analysis

The cambial zone tissue of each bulk was ground into powder in liquid N_2_ and dried for 24 h using the Modulyod-320 freeze dry system (Thermo Scientific). Metabolites were extracted from powder tissue and analyzed according to the methods described by Hoffman et al. (2010), with minor changes. Approximately 5 mg of dried tissue was mixed with 1 mL of a chloroform-methanol-water mix (6:2:2) containing stable isotope reference compounds [15 ng mL^−1^ each of (^13^C_3_)-myristic acid, (^13^C_4_)-hexadecanoic acid, (^2^H_4_)-succinic acid, (^13^C_5_, ^15^N)-glutamic acid, (^2^H_7_)-cholesterol, (^13^C_5_)-proline, (^13^C_4_)-disodium-ketoglutarate (^13^C_12_)-sucrose, (^2^H_4_)-putrescine, (^2^H_6_)-salicylic acid and (^13^C_6_)-glucose)]. The metabolite extraction proceeded using a vibration mill set to a frequency of 30 Hz s^−1^ for 3 min, with 3 mm tungsten carbide beads added to each extraction tube to increase the extraction efficiency. The extracts were then centrifuged for 10 min at 14.000 xg in an Eppendorf centrifuge (model 54178). After, 100 μL of each supernatant was transferred to a GC-vial and evaporated to dryness. The samples were then derivatized with 30 μL of methoxyamine hydrochloride (15 mg mL^−1^) in pyridine for 16 h at room temperature. Trimethylsilation was performed by adding 30 μL of n-methyl-n-(trimethylsilyl) trifluoroacetamide (MSTFA) with 1% TMCS to the samples and incubating them for 1 h at room temperature. After silylation, 30 μL of heptane was added. Samples were analyzed, according to Gullberg et al. (2004), using gas chromatography with time-of-flight mass spectrometry (GC-TOF/MS) together with blank control samples and a series of *n*-alkanes (C12–C40), which allowed retention indices to be calculated (Schauer et al., 2005). Three biological replicates with three technical replicates were used for each treatment. One microliter of each derivatized sample was injected splitless by a CTC Combi Pal Xt Duo autosampler (CTC Analytics AG, Switzerland) into an Agilent 7890A gas chromatograph equipped with a 30 m × 0.25 mm i.d. fused-silica capillary column with a chemically bonded 0.25 μm DB 5-MS UI stationary phase (J&W Scientific, Folsom, CA). The injector temperature was 260°C, the septum purge flowrate was 20 mL min^−1^ and the purge was turned on after 75 s. The gas flow rate through the column was 1 mL min^−1^, the column temperature was held at 70°C for 2 min, then increased by 15°C min^−1^ to 320°C, and held there for 4 min. The column effluent was introduced into the ion source of a Pegasus HT time-of-flight mass spectrometer (Leco Corporation, St. Joseph, MI, USA). The transfer line and the ion source temperatures were 250 and 200°C, respectively. Ions were generated by a 70 eV electron beam at an ionization current of 2.0 mA, and 20-30 spectra s^−1^ (30 spectras^−1^ run 1, 20 spectra s^−1^ run 2) were recorded in the mass range *m*/*z* 50−800. The acceleration voltage was turned on after a solvent delay of 290 s. The detector voltage was 1450-1490 V (1450 V run 1, 1490 V run 2). All non-processed MS-files from the metabolic analysis were exported into Chroma TOF 2.12 software (Leco Corporation), in which all manual integrations and metabolite identification were done. The identities of the compounds were determined by performing database searches based on the mass spectra and the compounds retention indices (RIs). The databases used were the NIST mass spectra library, an in-house database established by Umeå Plant Science Centre (UPSC), and the mass spectra library maintained by the Max Planck Institute in Golm, Germany. All data treatment procedures (smoothing, baseline correction and chromatogram alignment) were performed using custom scripts (Jonsson et al., 2005) in MATLAB. To compare the metabolite changes between summer and winter seasons the normalized data set (to tissue dry weight and internal standards) was Pareto scaled, log transformed and applied to multivariate and univariate analytical methods using the MetaboAnalyst software (Xia et al., 2012). The multivariate supervised classification method PLS-DA (Partial least squares-discriminant analysis) was carried out to discriminate between different groups (summer and winter). The variable importance in the projection (VIP) was used to rank the predictor variables, the metabolites, according to their contribution to the response in the respective PLS-DA model (Korn et al., 2010). VIP is a weighted sum of squares of the PLS loadings that takes into account the amount of explained Y-variance of each component (Xia and Wishart, 2011). PLS-DA model fit was evaluated using the *R*^2^ and *Q*^2^ cross-validation performance measures (Xia et al., 2012), both of which vary between 0 and 1. *R*^2^, the squared correlation coefficient between the dependent variable and the PLS-DA prediction, provides an indication of the “goodness of fit” (a value between zero and one, where one is a perfect correlation) from the model. *Q*^2^ provides an indication of “goodness-of-prediction” and is the averaged correlation coefficient between the dependent variable and the PLS-DA predictions. Differential metabolites were selected from the PLS-DA model using a combination of VIP value >1 and *P* ≤ 0.05, by the univariate unpaired, two-tailed Student's *t*-test.

### Carbohydrate extraction and HPLC analysis

Glucose, sucrose and fructose were extracted from cambial zone samples, three biological replicates with three technical replicates were used for each treatment. Tissues were grounded and freeze dried for 48 h, after 1 mL of water was added in 0.2 g of dry powder and samples were kept in bath (80°C) for 1 h. Then, samples were centrifuged for 10 min, 16.000 xg, the supernatant was recovered and stored at −4°C. Summer and winter samples were analyzed using a high-performance liquid chromatography (ICS 2500, HPLC Dionex) with amperometric detection (ED50) equipped with an autosampler AS50 (Dionex). Sugars were assigned according to the retention times of standards (sucrose, glucose and fructose). A Carbopac PA-1 column (4 × 250 mm, Dionex) and a guard Carbopac PA-10 column (4 × 50 mm, Dionex) were used. To identify statistical differences between summer and winter samples two-tailed Student's *t*-test (*P* ≤ 0.05) was performed.

## Results

### Changes in mRNA levels during summer and winter seasons

**Figure 1:**
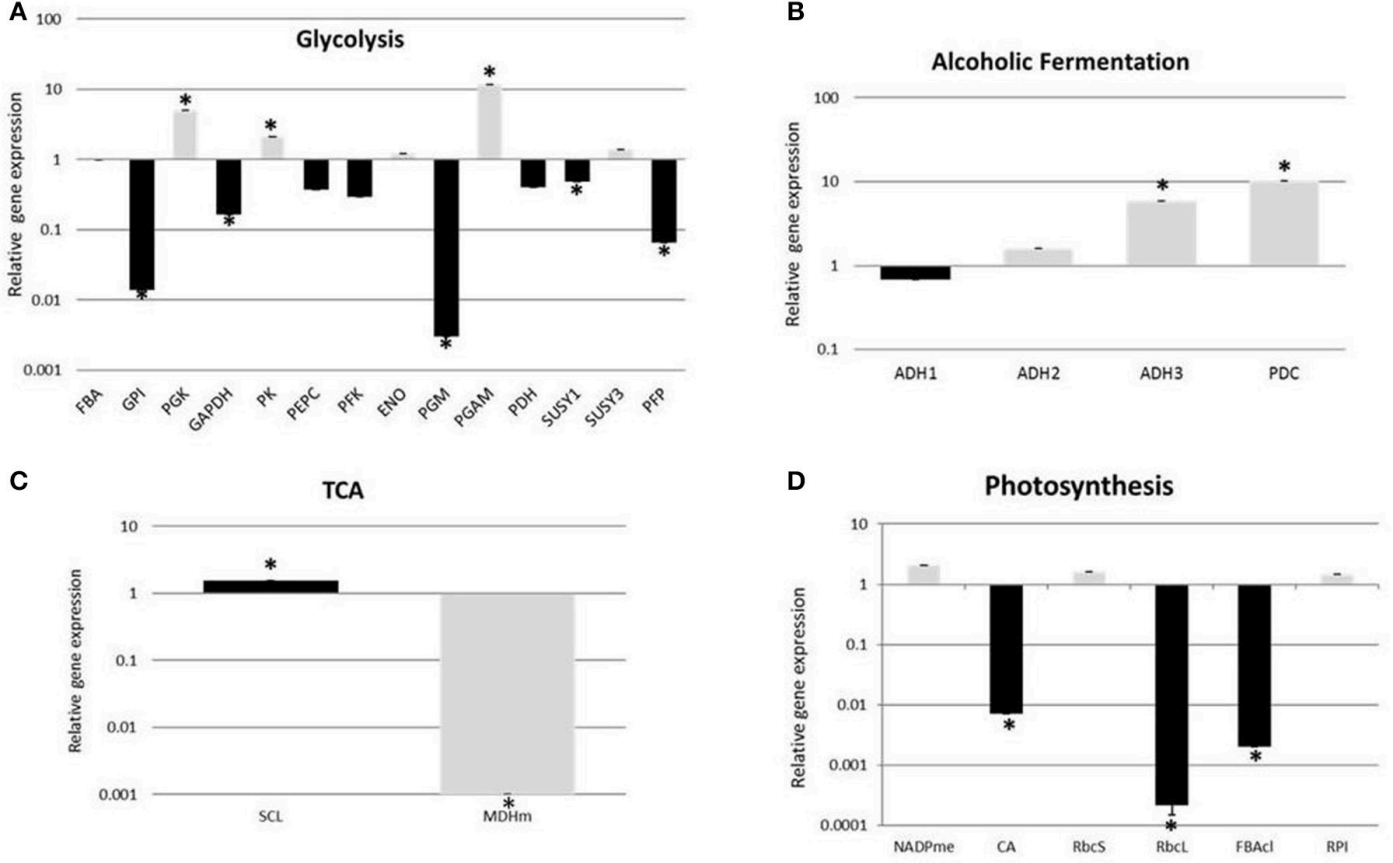
**Comparison of transcripts abundances of genes involved in primary metabolism: (A) glycolysis, (B) Alcoholic fermentation, (C) TCA cycle, and (D) Photosynthesis, in ***E. grandis*** cambial zone by RT-qPCR**. Data are expressed as log and winter values were used as a control. Expression was determined relative to alpha-tubulin and *MDH*, as described in Materials and Methods. Asterisks indicates genes that are significantly expressed (*P* ≤ 0.05). Abbreviations: *ALDO*, fructose bisphosphate aldolase; *GPI*, glucose 6 phosphate isomerase; *PGK*, phosphoglycerate kinase; *GAPDH*, glyceraldehyde 3 phosphate dehydrogenase; *PK*, pyruvate kinase; *PEPC*, phosphoenolpyruvate carboxylase; *PFK*, ATP-dependent phosphofructokinase; *ENO*, enolase; *PGM*, phosphoglucomutase; *PGAM*, phosphoglycerate mutase; *PDH*, pyruvate dehydrogenase; *SuSy1*, sucrose synthase 1; *SuSy3*, sucrose synthase 3; *PFP*, PPi-dependent phosphofructokinase; *ADH1*, alcohol dehydrogenase 1; *ADH2*, alcohol dehydrogenase 2; *ADH3*, alcohol dehydrogenase 3; *PDC*, pyruvate decarboxylase; *SCL*, succinyl-coa ligase; *MDHm*, malate dehydrogenase mitochondrial; *NADPme*, NADP-malic enzyme; *CA*, carbonic anhydrase; *RbcS*, rubisco small subunit; *RbcL*, rubisco large subunit; *FBAcl*, fructose bisphosphate aldolase chloroplastidial; *RPI*, ribose-5-phosphateisomerase. Three biological replicates, each with three technical replicates were analyzed per sample and error bars are standard errors of mean.

As a first step to understand the molecular mechanisms underlying the seasonal reorganization in the cambial zone, we examined the transcriptional pattern of 26 genes (Figure 1 and Supplementary Table 1) related to specific pathways of plant primary metabolism: glycolysis, ethanolic fermentation, TCA cycle and carbon fixation. These pathways were chosen because they are crucial to plant respiration, maintenance and ATP generation. Of the 14 genes analyzed from glycolysis, three were up-regulated in summer (*PGK*, phosphoglycerate kinase; PK, pyruvate kinase; *PGAM*, phosphoglycerate mutase) and five were up-regulated during winter (*GPI*, glucose-6-phosphate isomerase; *GAPDH*, glyceraldehyde-3-phosphate; *PGM*, phosphoglucomutase; Susy1, sucrose synthase 1; *PFP*, PPi-dependent phosphofructokinase; Figure 1A). *PGK* and *PGAM* genes catalyze subsequent reversible reactions in glycolysis while *PK* catalyzes an irreversible reaction. Among the five highly expressed genes in winter, four (*SUSY*1, *GPI, PFP*, and *GAPDH*) catalyze reversible reactions in glycolysis, whereas *PGM* catalyzes an irreversible reaction. *PFP, GPI*, and *PGM* participate in subsequent steps in glycolysis and were all up-regulated in winter. Considering the genes participating in ethanolic fermentation we analyzed three alcohol dehydrogenases (*ADH*) alternative transcripts, all of them from ADH class I, and one piruvate descarboxylase (*PDC*). Only *ADH*3 and *PDC* were statistically significant and both were up-regulated in summer (Figure 1B). In addition, the transcriptional levels of two TCA cycle genes (*SCL*, succinyl CoA ligase; *MDHm*, malate dehydrogenase) were analyzed (Figure 1C). *SCL* and *MDHm* showed opposing expression patterns, *SCL* was up-regulated in summer while *MDHm* was up-regulated in winter. Six genes involved in carbon fixation were analyzed (Figure 1D), with three being up-regulated during winter (*CA*, carbonic anhydrase; *RbcL*, rubisco large subunit; FBAcl, fructose bisphosphate aldolase).

### Protein profile in the cambial zone during summer and winter seasons

**Figure 2:**
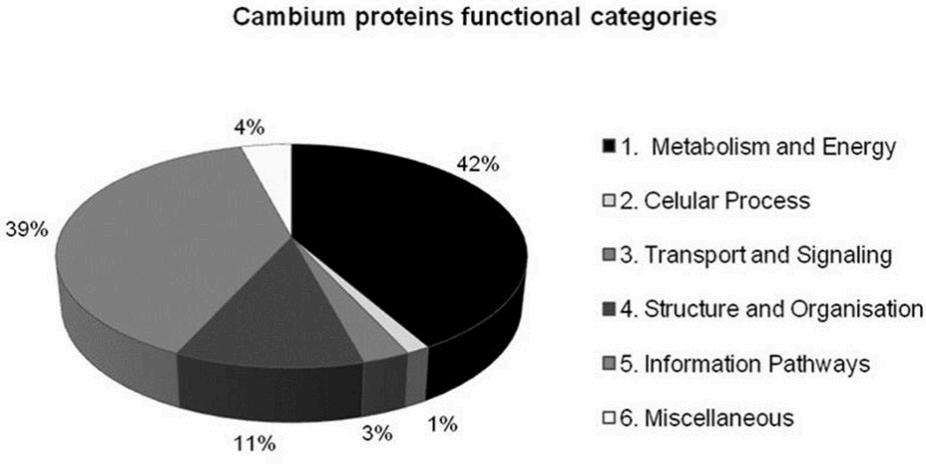
**Categorization of proteins identified in ***E. grandis*** cambial zone during summer and winter**.

Protein profile of cambial zone (summer vs. winter) were characterized using IEF-SDS-PAGE in conjunction with LC-MS/MS (Supplementary Figure 2). More than 400 proteins were detected by Image Master Platinum (GE Healthcare). Of these, 129 spots had significant (*P* ≤ 0.05) and reproducible changes in abundance between samples harvested in summer and winter. Among these proteins, ~60% (70 spots) were successfully identified (Table 1). From these identified proteins spots, 74.3% (52 spots) were up-regulated in summer and 25.7% (18 spots) in winter. The others proteins spots were disregarded because they did not show similarities when queried against *Eucalyptus* and NCBI databases or had scores lower than 70 (Perkins et al., 1999). Many analyzed peptides yielded hits from the same protein accession. Similar observations were made in other studies which provided possible explanations such as protein degradation and post-translational modification (Jiang et al., 2007; Shu et al., 2011; Galindo-González et al., 2012). The proteins identified were classified into six main biological categories (Figure 2 and Table 1) as described by Rison et al. (2000).

Proteins in the category of Metabolism and Energy were the most abundant (42%) and were distributed into five classes: Energetic Metabolism, Energy Transfer, Amino acid Metabolism, Nucleotide Metabolism and Secondary Metabolism. This category included mainly proteins related to glycolysis (aldolase, PGI, PGK, enolase, and GAPDH), ethanolic fermentation (ADH and PDC) and TCA cycle (citrate synthase, NADP-isocitrate dehydrogenase (NADP-IDH) and Pyruvate dehydrogenase). Most of these proteins were analyzed by RT-qPCR, showing similar expression patterns in many cases. The identification of proteins related to glycolysis, ethanolic fermentation and TCA cycle is interesting, because they are part of an interconnected pathway fulfilling the cambial region needs for ATP, NAD, NADH, and pyruvate for mitochondrial respiration (Celedon et al., 2007). In addition, three ADH isoforms were found, with ADH1 and 2 (spots 5 and 17) being highly expressed in summer, while ADH3 (spot 48) was highly expressed in winter. Another important protein identified was UGPase, for which two isoforms (spots 12 and 25) were mainly expressed in summer.

Contrary to results obtained with gene expression, we did not find proteins from carbon fixation pathways. This can be explained by limitations in 2-DE technique. The second most abundant category was the Information Pathway (39%), mainly represented by nine heat-shock proteins isoforms (six highly expressed in summer), besides two TCP-1/cpn60 chaperones (with opposite pattern of expression), two HSP20-like chaperones (highly expressed in winter) and three ATPases (highly expressed in summer). These are stress response proteins, acting in protein folding, assembly, translocation and degradation, maintenance of proteins functional conformation and preventing the aggregation of non-native proteins (Wang et al., 2004). Structure and Organization category was represented by proteins involved in the lignification process [caffeic acid O-methyltransferase (COMT) and caffeoyl CoA O-methyltransferase (CCoAOMT)] and proteins related to cytoskeleton formation (two alpha tubulins and one tubulin folding factor), all of them up-regulated in summer.

### Soluble sugars availability and metabolic changes in cambial zone during summer and winter

**Figure 3:**
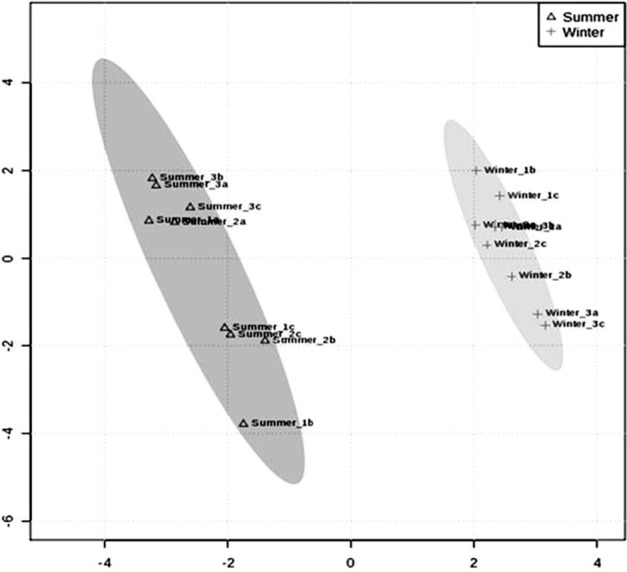
**PLS-DA score plots of eucalyptus cambial zone showing a significant separation (***R***^**2**^ = 0.99 and ***Q***^**2**^ = 0.98) between summer (square) and winter (circle) samples**. The x-axis represented the modeled co-variation and the y-axis is the predictive component. Component 1 and 2 contributes with 43.4 and 16.89%, respectively, of the total variance. Three biological replicates, each with three technical replicates were analyzed per sample.

To investigate the soluble sugar accumulation in response to seasonal changes we measured glucose, fructose and sucrose contents from summer and winter samples. The concentrations of all sugars were similar in both seasons. However, sucrose showed a higher concentration, compared to glucose and fructose, probably because it is the main sugar translocated in plants (Geiger, 2011). GC/TOF-MS was performed to identify changes in metabolites levels in the cambial zone due to seasonal variation. A total of 53 metabolites were identified and any differences between summer and winter samples were explored by multivariate and univariate methods (Supplementary Table 2). A PLS-DA model with 5 components was conducted to reveal the differences between the two groups. Based on this we found a clear separation between summer and winter groups (Figure 3). The two first principal components from PLS-DA accounted for 60% of the total variance and the model showed a significant *R*^2^ and *Q*^2^ values of 0.92 and 0.95, respectively. Based on VIPS >1 and *P* < 0.05 we found 19 differentially abundant metabolites between the summer and the winter groups (Table 2). The 19 metabolites were classified into five categories: amino acid (21%), fatty acid (5, 26%), organic acid (31, 57%), sugar (36, 84%), and unknown (5, 26%; Table 2). Most of the metabolites identified (68, 42%) were more abundant in summer samples, including all the amino acids.

**Table 2:** **Metabolites differentially abundant in cambial region**.

**Metabolite**	**Classification**	**VIP**	***p*-value**	**↑Abundance**
Glycine	Amino acid	1.4447	1.49E-07	Summer
Threonine	Amino acid	1.51038	3.92E-07	Summer
Valine	Amino acid	1.17622	1.05E-04	Summer
Serine	Amino acid	1.16592	1.67E-04	Summer
2,4,5-Trihydroxypentanoic acid	Fatty acid	1.87514	2.79E-06	Winter
Heptanoic acid	Fatty acid	1.01994	0.007938	Summer
Oleic acid	Fatty acid	1.1788	0.009486	Summer
Pipecolic acid	Organic acid	1.91088	8.37E-07	Winter
Maleic acid	Organic acid	1.2797	2.90E-04	Summer
Threonic acid-1,4-lactone	Organic acid	1.1084	3.28E-04	Summer
Quinic acid	Organic acid	1.0168	0.008177	Summer
Fumarate	Organic acid	1.010102	0.016264	Summer
Erythrose-4P	Sugar	2.07648	1.62E-08	Summer
Raffinose	Sugar	1.74866	2.20E-08	Winter
Glycerate	Sugar	1.50314	2.62E-06	Winter
Xylose	Sugar	1.12846	2.17E-05	Summer
Galactinol	Sugar	1.17872	1.37E-04	Winter
Xylitol	Sugar	1.37912	2.70E-04	Summer
UN_1649.7	Unknown	1.13912	6.98E-05	Winter

## Discussion

Growth is controlled by both, endogenous factors, such as plant genotype or physiological processes (Schrader et al., 2004), and by exogenous factors, such as temperature, light and water availability (Deslauriers and Morin, 2005). Regular fluctuations of these limiting factors promote a periodic rhythm in plant development, reproduced at the cambium level by an active and a dormant stage, even in tropical trees (Callado et al., 2013). Cambial activity is genetically controlled but the rhythm of new cells production is determined by environmental limitations (Kozlowski and Pallardy, 1997). However, cambial responses and variations in wood properties are often difficult to interpret and generalize, due to the complexity and co-variance of factors involved in controlling xylem development, including tree hormonal balance, carbon fixation and allocation and tree water status (Chaffey et al., 2002). Water availability is the major climate driver of wood production. As a consequence, months with lowest water availability are less favorable for cell expansion and can affect cell wall thickness thus, properties such as wood density may be markedly altered (Balducci et al., 2014; Wagner et al., 2016). In the current work we aimed to identify molecular changes in the cambial zone of *E. grandis* due to seasonal variations (summer and winter) observed trees growing in the southeast of Brazil. Summer is characterized as a raining season, in which cambium is actively growing. On the other hand, winter is a dry season and this low water availability is less favorable for cell expansion (Wagner et al., 2016). We hypothesized that the intense metabolic activity in cambium, during summer, is probably maintained by glycolysis and ethanolic fermentation because of the intense aerobic respiration that might deplete cambial O_2_ supplies. Thus, alcoholic fermentation is required to generate NAD^+^ so that glycolysis can produce limited amount of ATP for cellular maintenance until the re-establishment of oxidative phosphorylation (Drew, 1997). We also hypothesized that cambium seasonal variations observed in our work were mainly trigged by water availability in soil instead of low temperatures. We found that metabolic pathways related to primary metabolism are differentially regulated in summer and winter, resulting in different carbon partitioning in the two seasons. As we expected we found transcripts and proteins from alcoholic fermentation pathway highly abundant in summer, the season which coincides with the rainfall period. In this season, with plenty of available water, the tree is growing rapidly, the cambium zone is metabolically active and cell division must be occurring in high rates. Thus, NAD^+^ must be continuously regenerated by fermentative reactions, maintaining the primary metabolism in the cambial zone, corroborating our hypothesis. On the other hand, during winter, when it is dry, tree's growth rates diminished and cell walls are ticker, the flux of carbon seems to favoring carbohydrate accumulation, which will probably be required for the next growing season.

### Effects of seasonal variation in the gene regulation of metabolism, influencing carbon partioning in the cambial zone of *E. grandis*

Transcriptomic analyzes of different Eucalyptus tissues have been reported in an attempt to understand wood quality (Salazar et al., 2011, 2013), cold acclimation (Liu et al., 2014), frost tolerance (Fernández et al., 2015), and water stress (Thumma et al., 2012). In this study we analyzed a set of transcripts related to primary metabolism by RT-qPCR. We found differential transcription patterns for glycolytic genes in response to summer and winter seasons (Figure 1A). During winter the up-regulation of *PFP, GPI*, and *PGM* transcripts, which catalyze sequential step in glycolysis, might be indicating that the C flux is driving toward glucose-6-phosphate formation (G-6P), while the up-regulation of *SUSY*1 and *GAPDH* indicates that both enzymes are working to promote the formation of UDP-glucose (UDP-G) and glyceraldehyde-3-P (G-3P), respectively. In the summer condition, the up-regulation of *PGK, PGAM*, and *PK* suggests that C flux goes in the direction of pyruvate formation. As mentioned before, the final destination of the pyruvate formed in glycolysis, depends on the oxygen availability. In the presence of O_2_, pyruvate is completely oxidized to CO_2_ and water via aerobic respiration (Kimmerer and Stringer, 1988). However, when oxygen availability decreases, below the level at which oxygen becomes limiting for oxidative phosphorylation in TCA cycle, ethanolic fermentation is required. In our work *ADH3* and *PDC* were up-regulated in the cambial zone during summer (Figure 1B) indicating that fermentation might be high in order to supply glycolysis with NAD^+^ to maintain the supply of ATP, as we hypothesized. In addition, this energy produced might be utilized for cell division and biosynthesis of new cell wall in the cambium. Despite the importance of ethanolic fermentation for plant metabolism under O_2_ limitation, the TCA cycle is essential to maintain respiration even under anaerobic condition. The first study about alcohol dehydrogenase in tree stems were done by Kimmerer and Stringer (1988). They hypothesized that tree with thick bark would have greater ADH activity and ethanol content than thinner-barked trees of the same species, however, they did not observe any relation between bark thickness and ADH activity.

We found two transcripts of the TCA cycle (*SCL* and *MDHm*) differentially expressed and they showed opposite expression patterns (Figure 1C). The TCA cycle is a universal feature of the aerobic organism metabolism, thus eucalyptus probably has alternative transcripts of TCA cycle enzymes that are seasonally regulated. In RNA-seq experiments, Xu et al. (2014) found that 20.7% of highly expressed transcripts in the developing xylem of *Eucalyptus* were affected by alternative splicing events. More detailed studies are necessary to understand how seasonal changes regulate this cycle in woody plants. Glycolysis and TCA cycle pathways have several bypass reactions, allowing carbon flux to take different routes. The intermediates of central C metabolism also serve as precursor for synthesis of a plethora of primary and secondary metabolites (Plaxton and Podesta, 2006). Our results suggest that at the transcriptional level, cambial glycolysis and TCA cycle were differentially regulated, resulting in different carbon partitioning between the two seasons.

### Changes in proteins mainly related to primary metabolism in the cambial zone during summer and winter seasons

Through changes in protein profile, based on Student's *t*-test analysis (*P* ≤ 0.05), between summer and winter, we found five proteins from the glycolytic pathway (GPI, FBA, GAPDH, PGK and ENO), two proteins involved in ethanolic fermentation (ADH and PDC) and three proteins from TCA cycle (NADP - isocitrate dehydrogenase, citrate synthase and pyruvate dehydrogenase). Not all expression patterns of proteins were correlated to gene expression. These data suggest that in the cambial zone, a complex regulatory network occurred due to seasonal variation also at proteomic level.

In accordance with gene expression, during summer the cambial zone favors pyruvate production, which is distributed mainly to ethanolic fermentation. We found three ADH isoforms; ADH 1 and ADH 2 were highly abundant in summer while ADH 3 was highly abundant in winter. These ADH expression pattern suggest a temporal expression of the isoforms in the cambial zone and need to be further characterized. Another interesting finding were enzymes related to carbon metabolism, two UGPase isoforms, both highly expressed in summer. UGPase activity is related to cell wall synthesis and it promotes carbohydrate accumulation in storage tissues under low temperatures (Kleczkowski et al., 2004). Proteins of carbohydrate and energy metabolism have been reported in the stems of white spruce (Galindo-González et al., 2012), *Populus* (Plomion et al., 2006; Kalluri et al., 2009; Durand et al., 2011), *Eucalyptus* (Celedon et al., 2007; Leonardi et al., 2015), and *Pinus* (Gion et al., 2005). In summer samples, we also identified an upregulation of proteins involved in cell wall synthesis and cytoskeleton (Table 1). These data indicate that cell wall synthesis and deposition occurs during the growing season. Thus, remodeling of the cell wall could facilitate resilience against water deficit and low temperature conditions (Galindo-González et al., 2012).

### Seasonal variation of photosynthetic transcripts and proteins in *E. grandis* cambial zone

Although green leaves are regarded as the main source of photosynthates, the stem can be photosynthetically active (Wiebe, 1975; Pfanz et al., 2002; Aschan and Pfanz, 2003; Cernusak and Cheesman, 2015; Vandegehuchte et al., 2015). *Eucalyptus* and other trees experience the stem recycling photosynthesis (Ávila et al., 2014), in which the main function is to recycle some portion of the CO_2_ produced inside the stem as a result of respiratory activity in the woody tissue (Saveyn et al., 2010; Cernusak and Cheesman, 2015). Some authors have been investigating this issue from an ecological and physiological perspective (Berveiller et al., 2007; Eyles et al., 2009; Saveyn et al., 2010; Cernusak and Hutley, 2011), nevertheless photosynthesis in stem is receiving little attention at the molecular level. We checked the expression of six genes involved in carbon fixation and found three genes up-regulated in winter: CA, RbcL, and FBAcl (Figure 1D). Transcripts related to chloroplasts or photosynthetic activity, including RbcS, were found up-regulated in ray cambial cells of hybrid poplar (*Populus trichocarpa* T.&G. × *Populus deltoïdes* Marsh var. Boelare) compared to fusiform cambial cells (Goué et al., 2008). CA, RbcL and RbcS transcripts were previously identified in the cambial region of poplar trees (Hertzberg et al., 2001). It is interesting to note that we found transcripts of RbcL and RbcS, however, only RbcL expression was up-regulated in winter, suggesting that this subunit of Rubisco may be temporally regulated being highly required during winter. Rubisco is also considered to be an unconventional storage protein (Cooke and Weih, 2005). The up-regulation of RbcL may also indicate that perhaps, this subunit could play a storage function during winter, once trees accumulate carbon and nitrogen reserves that can be mobilized upon the next growing season. Similar result was observed by Galindo-González et al. (2012).

Contrary to our gene expression results, we did not find proteins from carbon fixation pathways. This can be explained by limitations in 2-DE technique such as, low detection sensitivity and linearity, limited loading capacity of gradient pH strips, poor solubility of membrane proteins among others (Monteoliva and Albar, 2004). CO_2_ fixation proteins such as sedoheptulose-bisphosphatase, ribose-5-phosphate isomerase and rubisco (large and small subunit) were found in *Picea glauca* stem (Galindo-González et al., 2012). Rubisco large subunit was found in eucalyptus cambium (Celedon et al., 2007) and both subunits were found in *Populus* cambial zone (Juan et al., 2006). Our findings are in accordance with these observations, giving molecular support for the concept that photosynthesis occurs in the stem. Nevertheless, more detailed studies are necessary to understand the role of photosynthetic transcripts and proteins in the cambial zone.

### Metabolic profiling changes in cambial zone during summer and winter

Most of the differentially abundant metabolites were more abundant in the summer season. This is the case for the amino acids glycine, serine, valine and threonine. In plants it has been proposed that the cytosolic conversion of serine into glycine, could be an important one-carbon source in plant C_1_ metabolism. It is possible that this metabolism is required during xylem maturation for the production of glycine, which is abundant in the cell wall proteins (Moreau et al., 2005). Oleic acid, was one of the three free fatty acids (FA) found. This saturated metabolite and others free FA were identified in the sapwood of *Pinus sylvelstris*, their levels were greatest in the beginning and end of the growing season (Saranpaa and Nyberg, 1987).

Xylose, xylitol and erythrose-4-phosphate (E4P) were abundantly found in summer. Xylitol, also called wood sugar, is made from xylose, which is found in the cell walls of most land plants (Nigam and Singh, 1995). Both sugars are important for the food and beverage industries (Nigam and Singh, 1995; Prakasham et al., 2009). We believed that xylose and xylitol were highly abundant in summer due to cell wall division and expansion during the tree active growth. Ko et al. (2011) found that xylose content increased in winter/dormancy stems of *Populus* trees. The E4P is an intermediate product of glucose oxidation through the pentose phosphate pathway (Bochkov et al., 2012). E4P and phosphoenolpyruvate (PEP) are precursors of the shikimate pathway (Zulak et al., 2008). This pathway provides an alternative route to aromatic compounds (Dewick, 2002) and is responsible to link the lignin biosynthesis to primary metabolism (Boerjan et al., 2003). Besides, the shikimate pathway is also related to the tree active growth (Larisch et al., 2012). Quinic acid, an organic acid precursor of aromatic amino acids (Minamikawa, 1976), was also highly abundant in summer. We also found the shikimic acid metabolite, but it did not show statistical differences between seasons. The organic acid metabolism is fundamental for several biochemical pathways, including energy production, formation of precursors for amino acid biosynthesis and at the whole plant level in modulating adaptation to the environment (López-Bucio et al., 2000). The organic compounds fumarate, maleic acid and threonic acid-1,4-lactone were more abundant during summer. Fumarate is a component of the TCA cycle and can be metabolized to yield energy and carbon skeletons of production of other compounds (Chia et al., 2000), its abundance in summer indicates that TCA cycle is also being required in addition to ethanolic fermentation. Pipecolic acid was the unique organic acid highly abundant during winter, it is a non-protein amino acid, homologous to proline that results from the lysine degradation, in plants. Pipecolic acid accumulation in higher plants seems to be closely related to metabolic responses induced by environmental stress (Moulin et al., 2002).

The three soluble sugars (glucose, sucrose and fructose) measured by HPLC were not statistically different between summer and winter. Since the cambial tissue is not recognized as a storage organ, carbohydrates present in this tissue are being supplied from the bark, which is the storage compartment. Similar results were reported in *Populus trichocarpa* (Ko et al., 2011). The authors measured soluble sugars from winter/dormancy and summer stem samples and observed a significant increase in total sugars content in the winter/dormancy stem. But, the concentrations of sucrose, fructose and glucose were similar in the two stem types from different seasons.

However, sugars identified by GC-MS were the metabolite category which was the most abundant in winter, having interesting metabolites such as glyceric acid, raffinose, and galactinol. Carbohydrate pools are mobilized in trees to supply respiration during low photosynthesis (Ögren, 2000), leaf development, bud-break, early spring growth (Barbaroux and Breda, 2002), when trees experiences stress such as water stress and pest damage (Canham et al., 1999). Different authors associate the carbohydrate accumulation with seasonal changes and physiological mechanisms in response to cold acclimation (Travert et al., 1997; Welling and Palva, 2006; Bonhomme et al., 2009; Turhan and Ergin, 2012). Raffinose and galactinol are known to act as cryoprotectors and were previously identified in the cambial region, accumulating in response to low temperatures (Guy et al., 2008; Ko et al., 2011; Plavcová et al., 2013). Raffinose levels increased in the cambium of mature conifer trees (*Picea abies Karst* and *Larix decidua Mil*) during winter, protecting cell membranes from damage during frost-induced dehydration by detoxifying radical oxygen species (ROS), that accumulates at low temperatures (Simard et al., 2013). On the other hand, both sugars have been hypothesized to be osmoprotectants in drought-stress conditions, and have frequently been implicated in drought response in plants (Taji et al., 2002; Nishizawa et al., 2008). Thus, we believed that in our work raffinose and galactinol might be accumulated during winter in response to diminished water availability instead of low temperatures. Raffinose and galactinol were some of the most highly accumulated metabolites in response to water-deficit conditions in *Populus balsamifera* leaves (Hamanishi et al., 2015). Deslauriers et al. (2014) found raffinose in low concentrations in the cambium and xylem of black spruce during the growing season. However, the authors reported an increase in raffinose levels in trees under water stress. All these results indicate a complex and dynamic metabolic change in cambial zone.

### Overview of the *E. grandis* primary metabolism in the cambial zone

**Figure 4:**
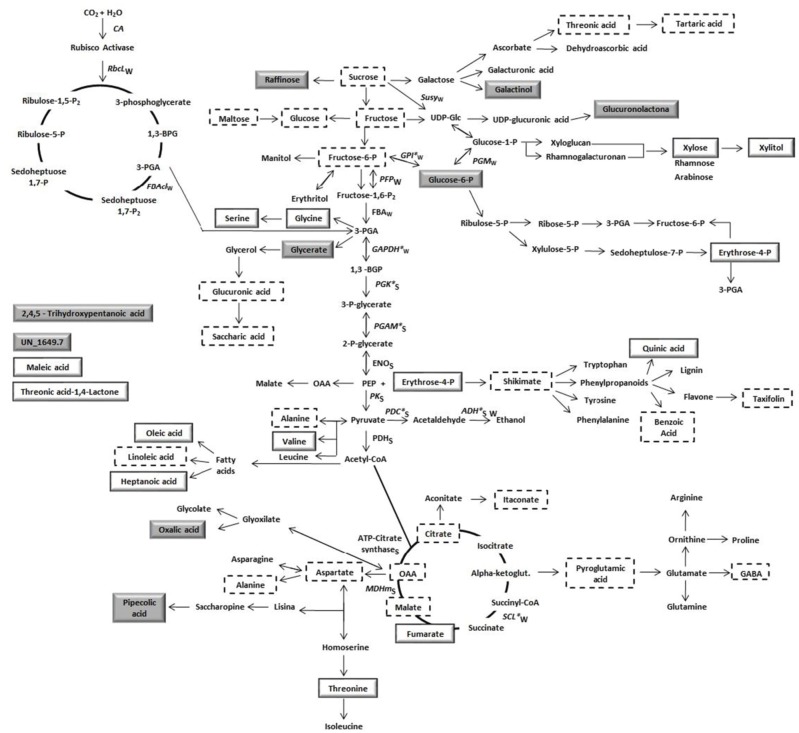
**Differentially abundant transcripts, proteins and metabolites involved in the cambial zone primary metabolism**. Transcripts are indicated by italic names and proteins are indicated by name. Subscribed letters S (summer) or W (winter) just below transcripts or proteins names indicates the season in which it was highly expressed. Asterisks in transcripts names indicates that the correspondent protein was also found as differentially expressed. Metabolites are indicated by boxes, metabolites in dashed boxes were detected in our work but were not significantly affected by seasonal changes (summer/winter). Metabolites highly abundant in summer or winter are indicates by white and gray boxes, respectively. RbcL, rubisco large subunit; FBAcl, fructose bisphosphate aldolase chloroplastidial; SuSy, sucrose synthase; GPI, glucose 6 phosphate isomerase; PGM, phosphoglucomutase; PFP, PPi-dependent phosphofructokinase; FBA, fructose bisphosphate aldolase citoplasmatic; GAPDH, glyceraldehyde 3 phosphate dehydrogenase; PGK, phosphoglycerate kinase; PGAM, phosphoglycerate mutase; ENO, enolase; PK, pyruvate kinase; PDC, pyruvate decarboxylase; ADH, alcohol dehydrogenase; PDH, pyruvate dehydrogenase; SCL, succinyl-coa ligase; MDHm, malate dehydrogenase mitochondrial; ATP-Citrate synthase.

Carbon partitioning is a fundamental process in plant physiology. At the most basic level, plants assimilate inorganic carbon and nitrogen into reduced compounds required for growth. At the molecular level the variety of produced metabolites is large and the pathways that they feed into are complex and interconnected (Tegeder and Weber, 2007). A more comprehensive knowledge about a biological process can be achieved by the analysis of transcripts, proteins and metabolites profiles. This could improve and facilitate the characterization of changes in levels of important compounds during cellular regulation. Using data from multiple platforms Srivastava et al. (2013) presented a system response of *Populus* to oxidative stress integrating data from analysis of the cambial region of wild-type controls and trees expressing high-isoeletric-point superoxide dismutase. However, most of the studies have focused on acquiring and integrating data at two omics levels (Alvarez et al., 2008; Hoffman et al., 2010; Galindo-González et al., 2012; Hamanishi et al., 2015; Tuttle et al., 2015; Yu et al., 2015). To have a better understanding about the dynamic changes occurring in the *E*. *grandis* cambial zone, due to summer and winter seasons, the RT-qPCR, proteomics and metabolomics data related to carbon metabolism were grouped (Figure 4). We observed differential abundance of molecules related to different pathways/metabolisms such as sugar, glycolysis, TCA cycle, amino acids, phenylpropanoids. We found significant changes in all levels (transcripts, proteins and metabolites), indicating a clear occurrence of a metabolic reconfiguration triggered by seasonal variation. This tight correlation between different levels of response in primary metabolism implies that transcriptional regulation plays an important role in carbon metabolism. The transcriptional and proteomic levels suggest different carbon partitioning between the two seasons. In summer, metabolism seems to favor pyruvate formation, which is metabolized by the fermentative enzymes. Thus, energy and reducing power are produced to supply the energetic demands required during the high metabolic activity of the season. Transcripts accumulation of genes involved in energy metabolism, cell division, differentiation and expansion were observed in the xylem tissues of *Pinus radiata* during its greater growth rate (Li et al., 2010). In this study, we showed a reconfiguration in the cambial zone metabolism of *E. grandis* due to seasonal changes in the tropic region. Although in the tropics plants do not experience extremely low temperatures similarly to those of temperate regions, our data indicate a reconfiguration in carbon partitioning from summer to winter. Different metabolic pathways were favored in each season. With this comprehensive dataset in hand, we can now explore the role of new genes/protein isoforms, mainly related to glycolysis and ethanolic fermentation, to study carbon partitioning during seasonal variation in trees.

## Author contributions

IB conceived of the study, carried out the experiments, analyzed the data and wrote the manuscript. DM assisted in the RT-qPCR analysis and manuscript revision. PL and TM assisted with metabolomics studies. CL conceived of the study, participated in its design and coordination, and reviewed the manuscript. All author's approved the final manuscript.

### Conflict of interest statement

The authors declare that the research was conducted in the absence of any commercial or financial relationships that could be construed as a potential conflict of interest.

## Supplementary material

Supplementary Figure 1 **Bark was removed and the tissues representing the cambial zone (differentiating xylem) were scrapped with a razor blade and immediately frozen with liquid nitrogen. (A)** Bark removal; **(B)** and **(C)** scrapping the tissue of cambial zone; **(D)** and **(E)** transversal section of eucalyptus wood before removal of the bark and after removal of the bark, respectively. Bars = 100 μm.

Supplementary Figure 2 **Representative 2-DE gels of eucalyptus cambial region during summer (A) and winter (B)**. Arrows indicate spots collected for protein identification.

Supplementary Table 1 **Primers used for RT-qPCR and the respective amplification size, efficiency (%) and P(H1) values**.

Supplementary Table 2 **Matrix containing the original metabolite data**.
