# Supplementary figures and images for: Seasonal Variation of Carbon Metabolism in the Cambial Zone of Eucalyptus grandis
Source: Front Plant Sci. 2016 Jun 28;7:932. doi: 10.3389/fpls.2016.00932 (PMC4923158; doi:10.3389/fpls.2016.00932)

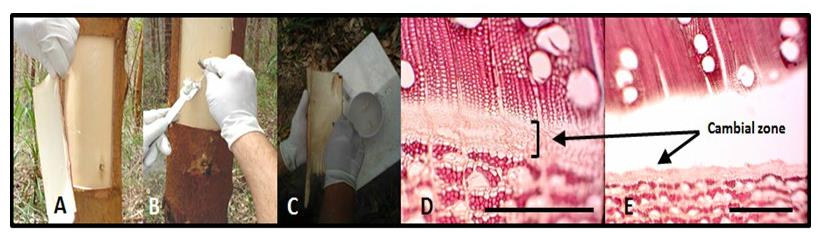

Supplement: Supplementary Figure 1 — Bark was removed and the tissues representing the cambial zone (differentiating xylem) were scrapped with a razor blade and immediately frozen with liquid nitrogen. (A) Bark removal; (B) and (C) scrapping the tissue of cambial zone; (D) and (E) transversal section of eucalyptus wood before removal of the bark and after removal of the bark, respectively. Bars = 100 μm. [file Image1.jpg]

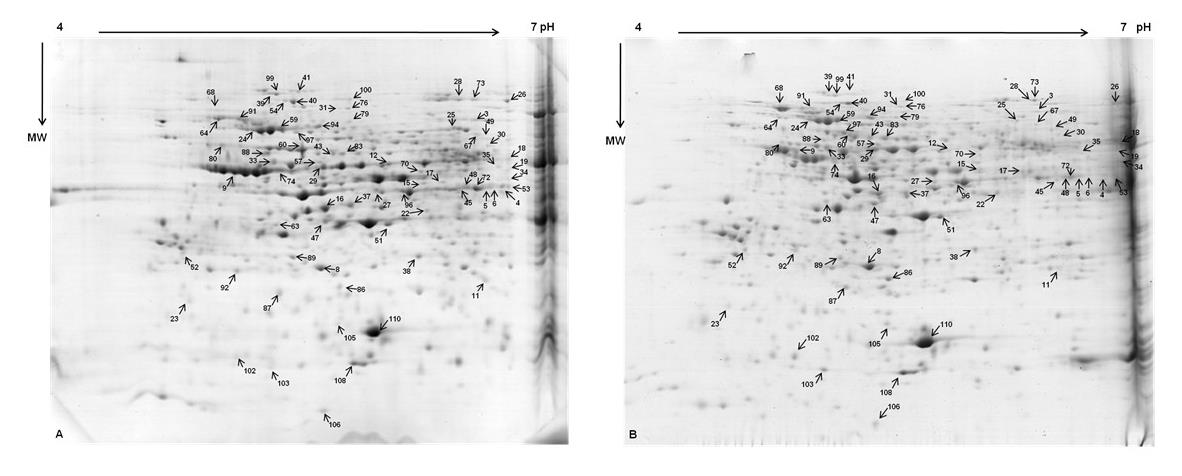

Supplement: Supplementary Figure 2 — Representative 2-DE gels of eucalyptus cambial region during summer (A) and winter (B). Arrows indicate spots collected for protein identification. [file Image2.jpg]
